# Supplementary material for: Candida auris skin tropism and antifungal resistance are mediated by carbonic anhydrase Nce103
Source: Nat Microbiol. 2025 Dec 23;11(2):461–75. doi: 10.1038/s41564-025-02189-z (PMC12872442; doi:10.1038/s41564-025-02189-z)
Supplement: Supplementary file 1 — Supplementary protocols. [file 41564_2025_2189_MOESM1_ESM.pdf]

nature microbiology

Supplementary information https://doi.org/10.1038/s41564-025-02189-z

Candida auris skin tropism and antifungal resistance are mediated by carbonic anhydrase Nce103

In the format provided by the authors and unedited

Supplementary Protocols

1. Proteomics

Several colonies of *C. auris* growing on YPD agar were picked and re-grown overnight in RPMI. Fungal suspensions were transferred to 50 ml fresh RPMI to reach OD_{600nm} of 0.1 in baffled flasks. After 5 hours incubation at 30 °C with agitation of 200 rpm, AMB was added at a final concentration of 0.5 or 0 µg/ml for an additional 2 hours. Yeast cells were pelleted and washed 3 times in cold PBS (Sigma-Aldrich). Cells were resuspended in tubes containing 300 mg of glass beads (Sigma-Aldrich) and 1 ml of Candida lysis buffer (1% sodium deoxycholate – SDC, 100 mM Tris-HCl, 150 mM NaCl, 1 mM PMSF, 1 mM EDTA 1 tablet/ 50 ml complete protease inhibitor), followed by bead beating (FastPrep-MPI) 6 m/s × 45 s × 3 times. The supernatant was collected by centrifuging through the small hole created at the bottom of the tube with a G26 needle tip. Protein was precipitated by 4 volume acetones, at -20 °C, overnight. Three biological replicates were performed for each experimental group.

The protein pellets were resuspended in 300 µl 4% (w/v) SDS, 100 mM Tris/HCl pH 8.5, and incubated at 95 °C for 5 min. The lysate was clarified by centrifugation at 16,000g for 10 min at 30 °C. The supernatant was then transferred to a new tube and protein concentrations were measured using a 600 nm protein assay kit (Pierce) with SDS compatibility reagents. 50 µg protein was reduced by adding 30 µl of 1 M dithiothreitol (DTT) and heated at 95 °C for 5 min. Samples were diluted with 9X 8 M urea in 100 mM Tris/HCl pH 8.5, transferred to the FASP filter, and centrifuged for 20 min at 12,000 g. After washing with 200 µl 8 M urea in 100 mM Tris/HCl pH 8.5 for 20 min at 120,00 g, 100 µl 50 mM iodoacetamide in 100 mM Tris/HCl pH 8.5 was added, vortex-mixed for 1 min and then incubated for 30 min in the dark at room temperature. After centrifugation for 10 min at 12,000 g, samples were washed twice with 200 µl 8 M urea in 100 mM Tris/HCl pH 8.5 and three times with 100 µl of 50 mM ammonium bicarbonate (ABC). The filter was transferred to a new collection tube and 40 µl of 50 mM ABC containing 1 µg trypsin platinum (Promega) was added and kept at 37 °C overnight. Digested peptides were collected by centrifuging for 15 min at 12,000 g. The filter was washed with 40 µl 50 mM ABC, centrifuged for 15 min at 12,000 g and pooled. The digested peptides were acidified with 10 µl 10% TFA and the peptides were desalted using C18 Stagetips[1] and MCX 96 well plates (Waters).

Tryptic peptides were separated on an Ultimate 3000 RSLC nano-flow chromatography system (Thermo-Fisher), using a pre-column for sample loading (Acclaim PepMap C18, 2 cm × 0.1 mm, 5 µm, Thermo-Fisher) and a C18 analytical column (Acclaim PepMap C18, 50 cm × 0.75 mm, 2 µm, Thermo-Fisher), applying a segmented linear gradient from 2 to 35% and finally 80% solvent B (80% acetonitrile, 0.1% formic acid; solvent A 0.1% formic acid) at a flow rate of 230 nl/min over 120 min. Eluted peptides were analyzed on an Exploris 480 Orbitrap mass spectrometer (Thermo-Fisher) coupled to the column with a FAIMS pro ion-source (Thermo-Fisher) using coated emitter tips (PepSep, MSWil) using the following settings. The mass spectrometer was operated in DDA mode

with two FAIMS compensation voltages (CV) set to -45 or -60 and 1.5 s cycle time per CV. The survey scans were obtained in a mass range of 350-1500 *m/z*, at a resolution of 60k at 200 m/z, and a normalized AGC target at 100%. The most intense ions were selected with an isolation width of 1.2 *m/z*, fragmented in the HCD cell at 28% collision energy, and the spectra recorded for max. 50 ms at a normalized AGC target of 100% and a resolution of 15k. Peptides with a charge of +2 to +6 were included for fragmentation, the peptide match feature was set to preferred, the exclude isotope feature was enabled, and selected precursors were dynamically excluded from repeated sampling for 45 seconds.

Raw data were split into single cv using FreeStyle 1.8 SP2 and processed using the MaxQuant software package (version 1.6.17.0)[2]. Data were mapped to the Uniprot *C. auris* reference proteome (version 2021.03, www.uniprot.org), as well as a database of most common contaminants (https://github.com/maxperutzlabs-ms/perutz-ms-contaminants). The search was performed with full trypsin specificity and a maximum of two missed cleavages at a protein and peptide spectrum match false discovery rate of 1%. Carbamidomethylation of cysteine residues were set as fixed, oxidation of methionine and N-terminal acetylation as variable modifications. For label-free quantification, we activated "match between runs" and "MaxLFQ", including normalization - all other parameters were used at default settings. Split raw files were processed in a single combined search, with each file assigned a fraction number corresponding to its CV value so as to ensure that match-between-runs was performed only within the same CV group.

MaxQuant output tables were further processed in R using Cassiopeia_LFQ (https://doi.org/10.5281/zenodo.5758974). Reverse database identification, contaminating proteins, protein groups identified only by a single modified peptide, protein groups with less than two quantitative values in one experimental group, and protein groups with less than 2 razor peptides were removed for further analysis. Missing values were replaced by randomly drawing data points from a normal distribution modelled on the whole dataset (data mean shifted by -1.8 standard deviations, width of distribution of 0.3 standard deviations). Differences between groups were statistically evaluated using the LIMMA package[3] at 5% FDR (Benjamini-Hochberg).

2. RNA isolation, qPCR and RNA-sequencing

Fungal cultures were centrifuged, and cell pellets were rapidly frozen in liquid nitrogen. Dry cell pellets were then stored at -80 °C for later use. Total RNA was extracted using the TRIZOL method, followed by DNase I treatment. Briefly, the fungal cells were resuspended in 1 ml of TRI Reagent (LabConsulting) and disrupted by FastPrep bead-beating (MP Biomedicals) with 300 mg glass beads at 6 m/s for 45 seconds × two times. Samples were centrifuged at 14,000 g for 10 min at 4 °C. The aqueous phase was further purified by adding 200 µl of chloroform, followed by precipitation with isopropanol at -20 °C for 30 min. RNA pellets were collected by centrifugation at 14,000 g for 20 min at 4 °C, pellets were washed with cold 70% ethanol before air-drying the RNA. Finally, RNA pellets were resuspended in 25 µl RNAse-free water (Invitrogen). Each sample equivalent to about 5 µg of

total RNA, underwent treatment with RNAse-free 10U of DNase I (Thermo Scientific) for 15 min in the presence of 50U of RiboLock RNase Inhibitor (Thermo Scientific). Subsequently, the RNA was purified by PCI extraction. The aqueous phase containing RNA was collected by ethanol precipitation with 30 mM sodium acetate at pH 5.3.

RNA quality and purification was checked with Nanodrop and conventional PCR-based quantification of *ACT1* mRNA. For quantitative PCR, first-strand cDNA was synthesized from RNA with Reverse Transcription System Kit (Promega). Subsequently, 15 ng cDNA was utilized for qPCR amplification, employing the 2x Luna Universal master mix (NEB). For competitive assays, gDNA was used directly for qPCR. The data was analyzed using the cloud-based system provided by Bio-Rad accessible at BR.io.

For RNA sequencing (RNA-seq) analysis, fungal cells grown overnight were diluted into 15 ml fresh YPD to reach OD_{600nm} of 0.1 in baffled flasks. Flasks were then incubated at 37 °C until reaching OD_{600nm} of 2.5. Fungal cells were collected by centrifugation, and RNA was isolated using TRIZOL method. At least three biological replicates were subjected to RNA-seq. RNA quality and integrity were evaluated with Bioanalyzer RNA 6000 Nanochip (Agilent Technologies). The library and sequencing were performed at the commercial Novogene Sequencing Facility (UK). Briefly, mRNA was enriched by poly-T oligo-attached magnetic beads followed by double-stranded cDNA library preparation. The quality-controlled RNA libraries were pooled and sequenced with 150-bp paired-end reads on the Illumina NovaSeq 6000 platform.

RNA-seq bioinformatics data analysis used a workflow established before[4] with some minor modification (https://github.com/kakulab/CSP2024). Briefly, quality of raw RNA-seq data was assessed by fastQC v0.11.9[5]. TrueSeq (Illumina) adapters were trimmed by cutadaptv2.8[6] (settings –interleaved -q 30), followed by read mapping on *C. auris* B8441 (AR387) genome assembly (Candida Genome Database version s01-m01-r22) using NextGenMap v0.5.5[7] (settings -b -Q 30). rRNA loci were removed by BEDtools v2.29.1[8] (settings: intersect -a -b -v). Then, Picard tools [9] from Broad Institute was used to remove duplicate reads (settings: MarkDuplicates REMOVE_SEQUENCING_DUPLICATES=true). HTSeq[10] was used for read counting in the union mode (settings htseq-count -f bam -r pos -t gene -i ID). Genomic annotation of *C. auris* B8841[11] version s01-m01-r22 was used. SAMtools v1.15.1[12] was used to prepare coverage files for Integrative Genomics Viewer[13] (IVG) visualization. Differential expression analysis was conducted using EdgeR v3.40.2[14], including the quasi-likelihood F-test (glmQLFTest) approach. Raw read counts were normalized using the TMM (Trimmed Mean of M-values) method via *calcNormFactors* in EdgeR. The false discovery rate (FDR) was controlled by adjusting p-values using the Benjamini–Hochberg correction. Principal component analysis (PCA) was conducted using CPM-normalized expression values obtained from the *cpm* function in EdgeR, applying TMM normalization (normalized.lib.size=TRUE). Genes with zero expression across all samples were removed, and PCA was conducted using the *prcomp* function in R (stats v3.4.1) with centering and scaling.

Visualization was done using ggbiplot. Gene ontology term enrichment analysis (enrichGO) and gene set enrichment analysis (GSEA) were performed with clusterProfiler[15] using annotation database retrieved from fungiDB[16] through AnnotationForce[17] package.

3. Sterol lipid quantification

Fungal cells from exponential growth phase were cultured in 15 ml RPMI from OD_{600nm} = 0.3 at 37 °C with shaking at 200 rpm. After 5 or 16 hours, cells were treated with 1 µg/ml AMB for additional two hours. Fungal cells were pelleted at 3000 rpm for 3 min, and washed 2 times with ice-cold PBS, before flash-freezing in liquid nitrogen and lyophilizing in a freeze dryer (LSL Secfroid - Lyolab BII, Switzerland). About 5 mg lyophilized powder from each fungal sample was transferred to a 2 ml safe lock tube containing a single metal bead (3 mm diameter, Qiagen 69997) and homogenized in a bead mill (Mixer Mill MM 400, Retsch) at maximum speed for 2 min. After resuspending in 1 ml of 2 M aqueous NaOH, samples were transferred to a 4 ml glass vial and incubated at 70 °C for 1 hour, with 10 seconds of vortex-mixing every 15 min. Vials were allowed to cool at room temperature to reach about 55 °C before transferring into 2 ml microcentrifuge tubes containing 650 µl methyl *tert*-butyl ether (M*t*BE, Roth – ROT.T175). We added 100 µl internal standard (IS) cholestane (Merck - C8003) to each tube. Tubes were first shaken vigorously by hand for 1 min before centrifugation at 10000g for 5 min at room temperature. The upper layer (roughly 550 µl) was transferred into a new microcentrifuge tube containing 35 mg Na_2SO_4 (Merck - 238597) and 5 mg PSA (Agilent - 5982-8382). The rest of cell lysate were extracted for the second time with another 750 µl of M*t*BE as in the first extraction step. Supernatants were combined, before obtaining a clean extract by centrifugation at 10,000 g for 5 min and transfer into a glass GC vial. Solvents were evaporated overnight in a chemical hood, and redissolved in 700 µl M*t*BE before adding 50 µl of a silylation reagent mixture 10:1 of *N*-methyl-*N*-trimethylsilyl-trifluoroacetamide (MSTFA; Macherey-Nagel - 701270.201) and *N*-trimethylsilyl-imidazole (TSIM; Macherey-Nagel, - 701310.201). The samples were analyzed in a Agilent 7820A gas chromatograph (GC) coupled to an Agilent quadrupole 5977B mass spectrometer (MS). Specific sterols and precursors were identified as their corresponding trimethylsilyl (TMS) ethers by mass spectra and relative retention times (RRT) [18]. The base peak of each sterol TMS ether was taken as a quantifier ion for determining the peak area for cholestane (IS) *m/z* 217 RRT 1.00, ergsta-5,8,22-trien-3β-ol (lichesterol) *m/z* 363 RRT 1.29, cholesta-8,24-dien-3β-ol (zymosterol) *m/z* 351 RRT 1.30, ergosta-5,7,22-trien-3β-ol (ergosterol) *m/z* 363 RRT 1.32, ergosta-7,22-dien-3β-ol *m/z* 343 RRT 1.34, ergosta-8,24(28)-dien-3β-ol (fecosterol) *m/z* 365 RRT 1.36, ergosta-5,7-dien-3β-ol *m/z* 365 RRT 1.40, ergosta-7,24(28)-dien-3β-ol *m/z* 343 RRT 1.40, ergost-7-en-3β-ol *m/z* 472 RRT 1.41, 4,4,14-trimethylcholesta-8,24(28)-dien-3β-ol (lanosterol) *m/z* 393 RRT 1.43, and 4-methylergosta-8,24(29)-dien-3β-ol (4-methylfecosterol) *m/z* 379 RRT 1.46. The sum of all detected peak areas of each sample was set as 100% and the percentage of each sterol was calculated[19,20].

4. Lipidomic analysis

Fungal cells were prepared similarly to the ergosterol quantification protocol. Briefly, cells were cultured for 5 hours, followed by a 2-hour treatment with/without 1 µg/ml AMB. After treatment, cells were washed three times with sterile dH_2O and flash-frozen in liquid nitrogen for subsequent analyses. Cell pellets were transferred to Precellys® tubes pre-filled with ceramic beads and homogenized in 155 mM ammonium acetate using a Precellys® Evolution tissue homogenizer (Bertin Technologies, Montigny-le-Bretonneux, France). Homogenization was carried out at 4 °C on ice through six cycles at 7500 rpm for 20 s each, with 30 s cooling intervals between cycles.

For lipid extraction, from each sample an aliquot corresponding to an OD_{600nm} of 0.01 was processed using a routine chloroform-based extraction protocol[21]. In brief, samples diluted in 600 µl cold water and mixed with 600 µl of $CHCl_3$:MeOH (1:2, v/v) were added. The samples were spiked with the internal standards Mouse SPLASH®, CerMix II, and cholesterol-d7 (Avanti Polar Lipids [Alabaster, USA]), sonicated in iced water for 10 s and then incubated for 1 h at 950 rpm and 4 °C. Subsequently, additional $CHCl_3$ and water were added, and samples were centrifuged at 10,000×g for 10 minutes at 4 °C. The lower organic phase was collected and gently dried using a nitrogen stream. For the analysis of glycerophospholipids (GP), glycerolipids (GL), and sterol esters (SE) direct infusion (DI) tandem mass spectrometry (MS/MS) was applied, while sphingolipids (SL) and sterols (ST) were measured using liquid chromatography (LC) coupled with MS/MS.

Lipid extracts for GP, GL, and SE were reconstituted in isopropanol (IPA):MeOH:$CHCl_3$ (4:2:1, v/v/v) containing 7.5 mM ammonium acetate and a volume of 12 µL was directly infused into an Exploris 240 Orbitrap mass spectrometer (ThermoScientific, Germany) via a TriVersa NanoMate ion source (Advion BioSciences, NY). The settings used for positive and negative mode were the following: ionization voltage +1.25 kV/ -1.25 kV, backpressure 0.95 psi, ion transfer capillary temperature 250 °C, S-Lens level of 60% and EASY-IC was enabled. Full MS spectra were acquired applying a resolution of 240,000 in both polarities. This was followed by a data-independent acquisition (DIA) for precursor masses at an interval of 1.001 Da using a resolution of 60,000 and a 1Da isolation width. Lipid identification was performed using LipidXplorer (version 1.2.8)[22], with Molecular Fragmentation Query Language (MFQL) queries tailored to match precursor and fragment ions. Resulting data were processed using lxPostman (LIFS).

For analysis of SL and ST, dried extracts were resuspended in BuOH:IPA:H_2O (8:23:69, v/v/v) with 5 mM phosphoric acid. From each sample, 5µl aliquots were injected into a Vanquish Flex UHPLC system (ThermoScientific, Germany) coupled to a QTRAP 6500+ mass spectrometer (AB Sciex, Germany) as reported before[23,24]. Chromatographic separation was performed using an Ascentis Express C18 column (150 mm × 2.1 mm, 2.7µm; Supelco, Bellefonte, PA) equipped with a guard cartridge (50 mm×2.1 mm, 2.7µm; Supelco), with column and autosampler temperatures maintained at 60 °C and 10 °C, respectively. Mobile phase A consisted of ACN:H_2O (60:40, v/v), and mobile phase B of IPA:ACN (90:10, v/v), both containing 10mM ammonium formate, 0.1% formic acid, and

5μM phosphoric acid. The flow rate was 0.5 ml/min, using the following 25-minute gradient: initial 30% B, held at 30% B from 0.0 to 2.0 min, 56.1% B at 3.0 min, 58.3% B at 4.0 min, 60.2% B at 5.5 min, 60.6% B at 7.0 min, 62.3% B 8.5 min, 64.0% B 10.0 min, 64.5% B 11.5 min, 66.2% B at 13.0 min, 66.9% B at 14.5 min, 100.0% B from 15.0 to 19.0 min, 5.0% B from 19.1 to 22.0 min, and 30 % B from 22.1 to 25.0 min. Prior to each injection, the needle was rinsed with a solvent matching the initial chromatographic conditions.

Data were acquired in positive ion mode using a Turbo V electrospray source with the following settings: curtain gas 30 arbitrary units, collision gas medium, ion spray voltage +5500 V, temperature 250 °C, ion source gas 1 40 arbitrary units, and ion source gas 2 65 arbitrary units. Scheduled multiple reaction monitoring (MRM) was employed using unit resolution for Q1 and Q3, a detection window of 2 min, and a scan time of 0.5 s. Inclusion lists were generated using LipidCreator (version 1.2.0)[25]. Data acquisition was done using Analyst (version 1.7.2; AB Sciex), and results were visualized and manually integrated in Skyline (version 22.2.0.312)[26]. Data processing and normalization were automated using KNIME (version 5.2.5)[27].

Signal intensities were normalized to the corresponding or most structurally similar internal standard. Triacylglycerols and cardiolipins were quantified based on precursor ion intensities, whereas all other lipid classes were quantified using the respective fragment ions. Data sets from shotgun and targeted analysis were combined to calculate molar percentages (mol%) of individual lipid species.

5. Genomic DNA isolation

Fungal cultures were centrifuged, and cells washed 2 times with sterile distilled water, followed by phenol - chloroform - isoamyl alcohol (PCI) extraction method. Cells were resuspended in Yeast Lysis Buffer (2% Triton X-100, 1% SDS, 100 mM NaCl, 1 mM EDTA, 10 mM Tris-Cl pH 8). The same amount of PCI (Sigma-Aldrich) was added before cell disruption by bead-beating (FastPrep-MPI) 6 m/s × 45 s × 2 times. Released DNA was washed with chloroform then precipitated from the aqueous phase with ethanol and acetate. DNA pellets were resuspended in water then treated with DNAse-free RNase A (Thermo Scientific - EN0531). DNA was reprecipitated with 40 mM ammonium acetate in ethanol at -20 °C and dissolved in nuclease free water (Invitrogen - 10977035).

6. Homology analysis

DNA sequences retrieved from CGD[11] were subjected to *blastn* from NCBI. Homology was searched on nucleotide collection (nr/nt) and whole-genome shotgun contigs (wgs) for *C. auris* (taxid:498019). Searching was done with the coding sequences for *RCA1, EFG1, NCE103*. Protein domain information was retrieved from the InterPro database[28].

Protein sequences of *Candida* spp. were retrieved from CGD[11]. *Blastp* searches were performed on *C. auris* proteins to find the best hits in target species. A reciprocal search against *C. auris* genome was conducted on *bastp* to identify orthologous genes. All sequences were put into *msa* package

version 1.32 for multiple sequence alignments[29]. Phylogenetic trees were constructed with neighbour-joining method using *nj* function in ape package version 5.7[30], followed by a bootstrap analysis with *boot.phylo*[30]. Synteny schemes were generated based on orthologous information obtained from the *Candida* Gene Order Browser (cgob.ucd.ie) and manually curated using data from *Candida* Genome Database.

Beta carbonic anhydrase structures of *C. albicans* (PDB: 6GWU and UniprotKB: Q5AJ71) and *Coccomyxa* (PDB: 3UCJ) were retrieved from the PDB and UniprotKB repositories. Computational homology models of *C. auris* Nce103 were generated using the SWISS-MODEL tool[31] using the coordinates for 6GWU (model 1) and 3UCJ (model 2).

7. Fluoresceine diacetate (FDA) uptake

Fungal cells from overnight cultures were grown to the exponential growth phase in YPD starting from an initial OD_{600nm} of 0.2. Cells were washed twice in FDA buffer (50 mM HEPES, pH 7.0 and 0.5 mM 2-deoxy-D-glucose; Sigma-Aldrich), followed by quantification in a CASY cell counter. Fungal cell suspensions were adjusted to 5×10^6 cells/ml in FDA buffer, before adding fluoresceine diacetate (Sigma-Aldrich) at a final concentration of 50 nM. Aliquots of 200 µl of cell suspensions with/without FDA were immediately transferred to a 96-well optical-bottom plate (ThermoScientific). The kinetics of FDA uptake was followed in real time by recording fluorescence every 5 min for 35 cycles at 37 °C in a Victor Nivo Microplate Reader at an excitation/emission setting of 485/535 nm[32].

References

1. Rappsilber, J., Mann, M. & Ishihama, Y. Protocol for micro-purification, enrichment, pre-fractionation and storage of peptides for proteomics using StageTips. *Nat. Protoc.* **2**, 1896–1906 (2007).
2. Tyanova, S., Temu, T. & Cox, J. The MaxQuant computational platform for mass spectrometry-based shotgun proteomics. *Nat. Protoc.* **11**, 2301–2319 (2016).
3. Ritchie, M. E. *et al.* limma powers differential expression analyses for RNA-sequencing and microarray studies. *Nucleic Acids Res.* **43**, e47–e47 (2015).
4. Jenull Sabrina *et al.* Transcriptomics and phenotyping define genetic signatures associated with echinocandin resistance in *Candida auris*. *mBio* **13**, e00799-22 (2022).
5. Andrews, S. FastQC: a quality control tool for high throughput sequence data. http://www.bioinformatics.babraham.ac.uk/projects/fastqc (2010).
6. Martin, M. Cutadapt removes adapter sequences from high-throughput sequencing reads. *EMBnet J.* **17**, 10–12 (2011).
7. Sedlazeck, F. J., Rescheneder, P. & von Haeseler, A. NextGenMap: fast and accurate read mapping in highly polymorphic genomes. *Bioinformatics* **29**, 2790–2791 (2013).
8. Quinlan, A. R. & Hall, I. M. BEDTools: a flexible suite of utilities for comparing genomic features. *Bioinformatics* **26**, 841–842 (2010).
9. Broad Institute. Picard Tools: A set of command line tools (in Java) for manipulating high-throughput sequencing (HTS) data and formats such as SAM/BAM/CRAM and VCF. Broad Institute (2010).
10. Putri, G. H., Anders, S., Pyl, P. T., Pimanda, J. E. & Zanini, F. Analysing high-throughput sequencing data in Python with HTSeq 2.0. *Bioinformatics* **38**, 2943–2945 (2022).
11. Skrzypek, M. S. *et al.* The Candida Genome Database (CGD): incorporation of Assembly 22, systematic identifiers and visualization of high throughput sequencing data. *Nucleic Acids Res.* **45**, D592–D596 (2017).
12. Danecek, P. *et al.* Twelve years of SAMtools and BCFtools. *GigaScience* **10**, giab008 (2021).
13. Robinson, J. T. *et al.* Integrative genomics viewer. *Nat. Biotechnol.* **29**, 24–26 (2011).
14. Robinson, M. D., McCarthy, D. J. & Smyth, G. K. edgeR: a Bioconductor package for differential expression analysis of digital gene expression data. *Bioinformatics* **26**, 139–140 (2010).
15. Wu, T. *et al.* clusterProfiler 4.0: A universal enrichment tool for interpreting omics data. *The Innovation* **2**, 100141 (2021).
16. Amos, B. *et al.* VEuPathDB: the eukaryotic pathogen, vector and host bioinformatics resource center. *Nucleic Acids Res.* **50**, D898–D911 (2022).
17. Carlson, M. & Pagès, H. AnnotationForge: Tools for building SQLite-based annotation data packages. *R Package Version* **1**, (2019).
18. Müller, C., Binder, U., Bracher, F. & Giera, M. Antifungal drug testing by combining minimal inhibitory concentration testing with target identification by gas chromatography–mass spectrometry. *Nat. Protoc.* **12**, 947–963 (2017).
19. Kühbacher Alexander *et al.* The cytochrome P450 reductase CprA is a rate-limiting factor for Cyp51A-mediated azole resistance in *Aspergillus fumigatus*. *Antimicrob. Agents Chemother.* **67**, e00918-23 (2023).
20. Müller, C. *et al.* Sterol composition of clinically relevant mucorales and changes resulting from posaconazole treatment. *Molecules* **23**, (2018).
21. Bligh, E. G. & Dyer, W. J. A rapid method of total lipid extraction and purification. *Can. J. Biochem. Physiol.* **37**, 911–917 (1959).
22. Herzog, R. *et al.* LipidXplorer: A software for consensual cross-platform lipidomics. *PLOS ONE* **7**, e29851 (2012).
23. Troppmair, N. *et al.* Accurate sphingolipid quantification reducing fragmentation bias by nonlinear models. *Anal. Chem.* **95**, 15227–15235 (2023).
24. Peng, B. *et al.* A comprehensive high-resolution targeted workflow for the deep profiling of sphingolipids. *Anal. Chem.* **89**, 12480–12487 (2017).
25. Peng, B. *et al.* LipidCreator workbench to probe the lipidomic landscape. *Nat. Commun.* **11**, 2057 (2020).
26. Marsh, A. N. *et al.* Skyline for small molecules: a unifying software package for quantitative metabolomics. *J. Proteome Res.* **21**, 289–294 (2022).
27. Berthold, M. R. *et al.* KNIME: The Konstanz information miner. in *Data Analysis, Machine Learning and Applications* (eds. Preisach, C., Burkhardt, H., Schmidt-Thieme, L. & Decker, R.) 319–326 (Springer Berlin Heidelberg, Berlin, Heidelberg, 2008).
28. Blum, M. *et al.* InterPro: the protein sequence classification resource in 2025. *Nucleic Acids Res.* **53**, D444–D456 (2025).
29. Bodenhofer, U., Bonatesta, E., Horejš-Kainrath, C. & Hochreiter, S. msa: an R package for multiple sequence alignment. *Bioinformatics* **31**, 3997–3999 (2015).
30. Popescu, A.-A., Huber, K. T. & Paradis, E. ape 3.0: New tools for distance-based phylogenetics and evolutionary analysis in R. *Bioinformatics* **28**, 1536–1537 (2012).
31. Waterhouse, A. *et al.* SWISS-MODEL: homology modelling of protein structures and complexes. *Nucleic Acids Res.* **46**, W296–W303 (2018).
32. Shivarathri Raju *et al.* Comparative transcriptomics reveal possible mechanisms of amphotericin B resistance in *Candida auris*. *Antimicrob. Agents Chemother.* **66**, e02276-21 (2022).
