# Supplementary material for: Candida auris skin tropism and antifungal resistance are mediated by carbonic anhydrase Nce103
Source: Nat Microbiol. 2025 Dec 23;11(2):461–75. doi: 10.1038/s41564-025-02189-z (PMC12872442; doi:10.1038/s41564-025-02189-z)
Supplement: Supplementary file 2 — Reporting Summary [file 41564_2025_2189_MOESM2_ESM.pdf]

nature portfolio

Corresponding author(s): Karl Kuchler
Last updated by author(s): Oct 4, 2025

Reporting Summary

Nature Portfolio wishes to improve the reproducibility of the work that we publish. This form provides structure for consistency and transparency in reporting. For further information on Nature Portfolio policies, see our Editorial Policies and the Editorial Policy Checklist.

Statistics

For all statistical analyses, confirm that the following items are present in the figure legend, table legend, main text, or Methods section.

n/a	Confirmed	
☐	☒	The exact sample size (*n*) for each experimental group/condition, given as a discrete number and unit of measurement
☐	☒	A statement on whether measurements were taken from distinct samples or whether the same sample was measured repeatedly
☐	☒	The statistical test(s) used AND whether they are one- or two-sided<br>*Only common tests should be described solely by name; describe more complex techniques in the Methods section.*
☒	☐	A description of all covariates tested
☐	☒	A description of any assumptions or corrections, such as tests of normality and adjustment for multiple comparisons
☐	☒	A full description of the statistical parameters including central tendency (e.g. means) or other basic estimates (e.g. regression coefficient) AND variation (e.g. standard deviation) or associated estimates of uncertainty (e.g. confidence intervals)
☐	☒	For null hypothesis testing, the test statistic (e.g. *F*, *t*, *r*) with confidence intervals, effect sizes, degrees of freedom and *P* value noted<br>*Give P values as exact values whenever suitable.*
☒	☐	For Bayesian analysis, information on the choice of priors and Markov chain Monte Carlo settings
☒	☐	For hierarchical and complex designs, identification of the appropriate level for tests and full reporting of outcomes
☐	☒	Estimates of effect sizes (e.g. Cohen's *d*, Pearson's *r*), indicating how they were calculated

Our web collection on statistics for biologists contains articles on many of the points above.

Software and code

Policy information about availability of computer code

Data collection	BD FACSDiva™ Software for Flow Cytometry. For lipidomics, LipidCreator (version 1.2.0), Analyst (version 1.7.2; AB Sciex), Skyline (version 22.2.0.312) and KNIME (version 5.2.5) were used.
Data analysis	All software and code used in this study are described in the Methods section, Supplementary Table 4, and Supplementary Information. Briefly, MaxQuant software (version 1.6.17.0), Cassiopeia_LFQ (https://doi.org/10.5281/zenodo.5758974), and the LIMMA R package were used for proteomics analysis. For RNA-seq analysis, the following tools were utilized: FastQC v0.11.9, NextGenMap v0.5.5, BEDtools v2.29.1, HTSeq v0.11.2, SAMtools v1.15.1, and EdgeR v3.40.2. R packages including msa v1.32, ape v5.7, and ggtree v3.9.1 were used for multiple sequence alignment and neighbor-joining tree construction. GraphPad Prism v9.0, along with R packages rstatix v0.7.2 and ggplot2 v3.4.2, were used for statistical analysis and data visualization. FlowJo v10.8 was used for flow cytometry data analysis. Code for RNA sequencing analysis and data integration is available at https://github.com/kakulab/CSP2024.

For manuscripts utilizing custom algorithms or software that are central to the research but not yet described in published literature, software must be made available to editors and reviewers. We strongly encourage code deposition in a community repository (e.g. GitHub). See the Nature Portfolio guidelines for submitting code & software for further information.

Data

Policy information about availability of data

All manuscripts must include a data availability statement. This statement should provide the following information, where applicable:

- Accession codes, unique identifiers, or web links for publicly available datasets
- A description of any restrictions on data availability
- For clinical datasets or third party data, please ensure that the statement adheres to our policy

Data Availability
The proteomics data was deposited to the ProteomeXchange Consortium via the PRIDE partner repository113 with the dataset identifier PXD048342. RNA-seq data are available from the Gene Expression Omnibus (GEO) database with the accession number GSE253332. Lipidomics datasets are provided in supplementary data. Data used for figure generation are deposited as a Source Data file. Correspondence and requests for materials should be addressed to Karl Kuchler (kuchlerkarl1@gmail.com).
Code Availability
The code for RNA-seq analysis workflow was deposited on Github available through https://github.com/kakulab/CSP2024. The workflow for processing MaxQuant output tables was deposited at https://doi.org/10.5281/zenodo.5758974.

Research involving human participants, their data, or biological material

Policy information about studies with human participants or human data. See also policy information about sex, gender (identity/presentation), and sexual orientation and race, ethnicity and racism.

Reporting on sex and gender	Human skin samples were obtained from healthy adult female donors.
Reporting on race, ethnicity, or other socially relevant groupings	Not applicable.
Population characteristics	Healthy adult female donors
Recruitment	Participants were recruited from the patient pool of the Department of Plastic and Reconstructive Surgery, Medical University of Vienna, Währinger Gürtel 18-20, 1090 Vienna, Austria. Before their surgery, all participants were informed about the relevance of this study by the investigating physician of the Department of Plastic and Reconstructive Surgery. There were no additional risks or burdens for the study participants.<br>Human skin biopsy samples were obtained from three available donors. Donors were included based on availability, without random selection or specific covariate criteria (e.g., age, sex, medical history). Given the small number and the use of samples solely for ex vivo colonization assays, potential selection bias is not expected to impact the study outcomes.
Ethics oversight	Abdominal human skin samples were obtained from anonymous healthy adult female donors with approved consent following the Declaration of Helsinki and ethics committee approvals of the Medical University of Vienna (ECS 1969/2021).

Note that full information on the approval of the study protocol must also be provided in the manuscript.

Field-specific reporting

Please select the one below that is the best fit for your research. If you are not sure, read the appropriate sections before making your selection.

☒ Life sciences ☐ Behavioural & social sciences ☐ Ecological, evolutionary & environmental sciences

For a reference copy of the document with all sections, see nature.com/documents/nr-reporting-summary-flat.pdf

Life sciences study design

All studies must disclose on these points even when the disclosure is negative.

Sample size	Sample sizes of experimental groups were chosen based on community standards (e.g., 3 experimental repeats if not otherwise specified; at least 5 mice per group were used for in vivo experiments).
Data exclusions	No data were excluded from analyses workflows
Replication	Replication in the form of biological repeats is reported in the manuscript. For the study of human skin colonization, three skin biopsy samples were obtained from each of three different donors. Mouse experiments were replicated independently at least twice, and results were pooled. For in vitro experiments, at least three biological replicates were performed unless otherwise specified. In some cases, two biological or independent replicates were conducted instead of three due to the inclusion of multiple independent clinical strains; in such cases, conclusions were also confirmed by additional experiments (e.g., genetic ablation in addition to phenotypic assays).

Randomization	Female mice were randomly assigned to study groups. Skin biopsy samples and mice were randomly selected for infection with wild-type or mutant fungal strains.
Blinding	Blinding was not applied during allocation, outcome assessment, data collection, or analysis, as no potential sources of bias were identified.

Reporting for specific materials, systems and methods

We require information from authors about some types of materials, experimental systems and methods used in many studies. Here, indicate whether each material, system or method listed is relevant to your study. If you are not sure if a list item applies to your research, read the appropriate section before selecting a response.

Materials & experimental systems

n/a	Involved in the study	
☒	☐	Antibodies
☒	☐	Eukaryotic cell lines
☒	☐	Palaeontology and archaeology
☐	☒	Animals and other organisms
☒	☐	Clinical data
☒	☐	Dual use research of concern
☒	☐	Plants

Methods

n/a	Involved in the study	
☒	☐	ChIP-seq
☐	☒	Flow cytometry
☒	☐	MRI-based neuroimaging

Animals and other research organisms

Policy information about studies involving animals; ARRIVE guidelines recommended for reporting animal research, and Sex and Gender in Research

Laboratory animals	Adult wild-type female C57BL/6J mice (Mus musculus) were housed in specific pathogen-free conditions, with controlled temperature (20-22 °C) and humidity (45-65%), in a 12-h light/dark cycle at the Max Perutz Labs Vienna. Mice breeding and maintenance was in accordance with ethical animal license protocols complying with the current Austrian law. Animals used for experiments were wild-type C57BL/6J mice aged 8 to 14 weeks at the start of experiments.
Wild animals	No wild animals were used.
Reporting on sex	All skin experiments were performed with female mice. Tail vein infections were performed in both female and male mice.
Field-collected samples	No field-collected samples were used.
Ethics oversight	Animal experiments adhered to ethical approval from the ethics committee of the Medical University of Vienna and the Federal Ministry of Science and Research, Vienna, Austria (BMBWF-66.009/0436-V/3b/2019).

Note that full information on the approval of the study protocol must also be provided in the manuscript.

Plants

Seed stocks	Not applied
Novel plant genotypes	Not applied
Authentication	Not applied

Flow Cytometry

Plots

Confirm that:

☒ The axis labels state the marker and fluorochrome used (e.g. CD4-FITC).

☐ The axis scales are clearly visible. Include numbers along axes only for bottom left plot of group (a 'group' is an analysis of identical markers).

☐ All plots are contour plots with outliers or pseudocolor plots.

☐ A numerical value for number of cells or percentage (with statistics) is provided.

Methodology

Sample preparation	Sample preparations are described in the Methods section. Briefly, fungal cells were collected, washed before subjecting to flow cytometry analysis.
Instrument	BD LSRFortessa™ Cell Analyzer
Software	BD FACSDiva™ Software, FlowJo™ Software 10.8
Cell population abundance	Approximately 80-95% of fungal cells were obtained after excluding cell debris.
Gating strategy	Fungal cells were gated using FCS-A and SSC-A parameters, followed by single-cell gating using FCS-A and FCS-H. As only a single color was used, complex gating strategies were not required.

☐ Tick this box to confirm that a figure exemplifying the gating strategy is provided in the Supplementary Information.
